# Can “Rover” help with mental health during the COVID-19 pandemic? Results from the Canadian Longitudinal Study on Aging (CLSA)

**DOI:** 10.3389/fpsyt.2022.961067

**Published:** 2022-09-29

**Authors:** Ryan S. Falck, Teresa Liu-Ambrose, Matthew Noseworthy, Susan Kirkland, Lauren E. Griffith, Nicole E. Basta, Jacqueline M. McMillan, Parminder Raina

**Affiliations:** ^1^Aging, Mobility and Cognitive Neuroscience Laboratory, Department of Physical Therapy, University of British Columbia, Vancouver, BC, Canada; ^2^Centre for Hip Health and Mobility, University of British Columbia, Vancouver, BC, Canada; ^3^Djavad Mowafaghian Centre for Brain Health, University of British Columbia, Vancouver, BC, Canada; ^4^Department of Community Health and Epidemiology and Division of Geriatric Medicine, Dalhousie University, Halifax, NS, Canada; ^5^Department of Health Research Methods, Evidence, and Impact, Faculty of Health Sciences, McMaster University, Hamilton, ON, Canada; ^6^Cross-Departmental Research Institute for Advancing the Science of Aging, McMaster Institute for Research on Aging, McMaster University, Hamilton, ON, Canada; ^7^Department of Epidemiology, Biostatistics and Occupational Health, School of Population and Global Health, McGill University, Montreal, QC, Canada; ^8^Department of Medicine, University of Calgary Cumming School of Medicine, Calgary, AB, Canada

**Keywords:** pet ownership, mental health, COVID-19, Canadian Longitudinal Study on Aging, depression, anxiety

## Abstract

COVID-19 has negatively affected the mental health and well-being of adults, and thus it is important to examine potential factors which may influence mental health during the pandemic. We thus examined the association between pet ownership and depression/anxiety symptoms based on mental health disorder status during the COVID-19 pandemic. We included 12,068 cognitively healthy participants (45–86 years at study entry) from the Canadian Longitudinal Study on Aging (CLSA) comprehensive cohort who completed the first follow-up ([FU1]; 2015–2018), and COVID-19 Survey entry (April–May 2020) and exit (September-December 2020). Participants self-reported at FU1 if they owned a pet (yes/no). Participants were dichotomized as with or without a mental health disorder based on self-reported diagnosis of depression, anxiety, or mood disorders at baseline assessment (2011–2015) or FU1. Depressive symptoms were indexed using the 10-item Center for Epidemiological Studies Depression Scale (CESD-10) at FU1, and COVID-19 entry/exit surveys. Anxiety symptoms were assessed using the General Anxiety Disorder Questionnaire (GAD-7) at COVID-19 entry/exit surveys. Final models adjusted for age, sex, body mass index, income, education, living status, smoking status, relationship status, and alcohol intake. Forty-percent of participants owned a pet at FU1. Among those without a mental health disorder, there were no significant differences in CESD-10 between participants who owned pets compared with those without pets. For people with a mental health disorder, pet owners had higher CESD-10 (estimated mean difference range: 0.56–1.02 points; *p* < 0.05) and GAD-7 scores (estimated mean difference range: 0.28–0.57 points; *p* < 0.05) at both COVID-19 entry and exit surveys. Among people with mental health disorders, pet ownership was associated with poor mental health symptoms during April 2020 to December 2020 of the COVID-19 pandemic.

## Introduction

Coronavirus disease 2019 (COVID-19) has negatively affected the mental health and well-being of adults (1–3). Public health measures to combat the spread of the virus—such as self-isolation and quarantine—have affected usual activities and routines, which may lead to poorer mental health (4, 5). It is thus important to examine factors which may influence mental health during the pandemic.

People living with mental health disorders have experienced poorer mental health during the pandemic. A history of major depression, generalized anxiety disorder, or mood disorders (i.e., a set of psychological disorders characterized by the elevation or lowering of an individual's mood, such as bipolar disorder) is associated with ~0.2–0.5 SD increase in depressive and anxiety symptoms over and above pre-pandemic levels (6). Thus, it is critical to identify mitigating strategies for this population (7).

Pet ownership may provide a range of mental health benefits (8, 9). Owning a pet is a stress-relieving mechanism and can be used as a positive coping strategy (10). Interacting with pets reduces stress (11), and promotes better physical and mental well-being (12). Pet ownership improves quality of life, facilitates social and community interaction (13), and participation in pet-related activities like dog walking can decrease isolation and loneliness (14). However, it is unclear how pets have impacted mental health during the COVID-19 pandemic, nor is it clear whether pet ownership provides comparable benefits for people with mental health disorders (15).

The purpose of this study was thus to examine the association between pet ownership and depression/anxiety symptoms during the COVID-19 pandemic among people with and without mental health disorders. We hypothesized that pet ownership would be associated with less depression/anxiety symptoms during the pandemic in both people with and without mental health disorders.

## Methods

We conducted a longitudinal analysis using data from the Canadian Longitudinal Study on Aging (CLSA) comprehensive cohort (*N* = 30,097) from first follow-up ([FU1]; 2015–2018), and the COVID-19 Survey (April 2020–December 2020)—a sub-study of the CLSA among 28,457 participants from both the Comprehensive and Tracking cohorts. Detailed methods of the CLSA baseline, FU1, and COVID-19 Survey can be found elsewhere (2, 16, 17). All research conducted as part of the CLSA abides by the requirements of the Canadian Institutes of Health Research and relevant institutions for ethical conduct and privacy protection in health research. We received ethics approval from the University of British Columbia's Clinical Research Ethics Board. All subjects gave written informed consent.

### Inclusion criteria

The CLSA is a large, national, longitudinal study of Canadian women and men who were 45 to 85 years when recruited in 2010–2015. Information on the CLSA inclusion and exclusion criteria can be found elsewhere (16, 18).

**Figure 1:**
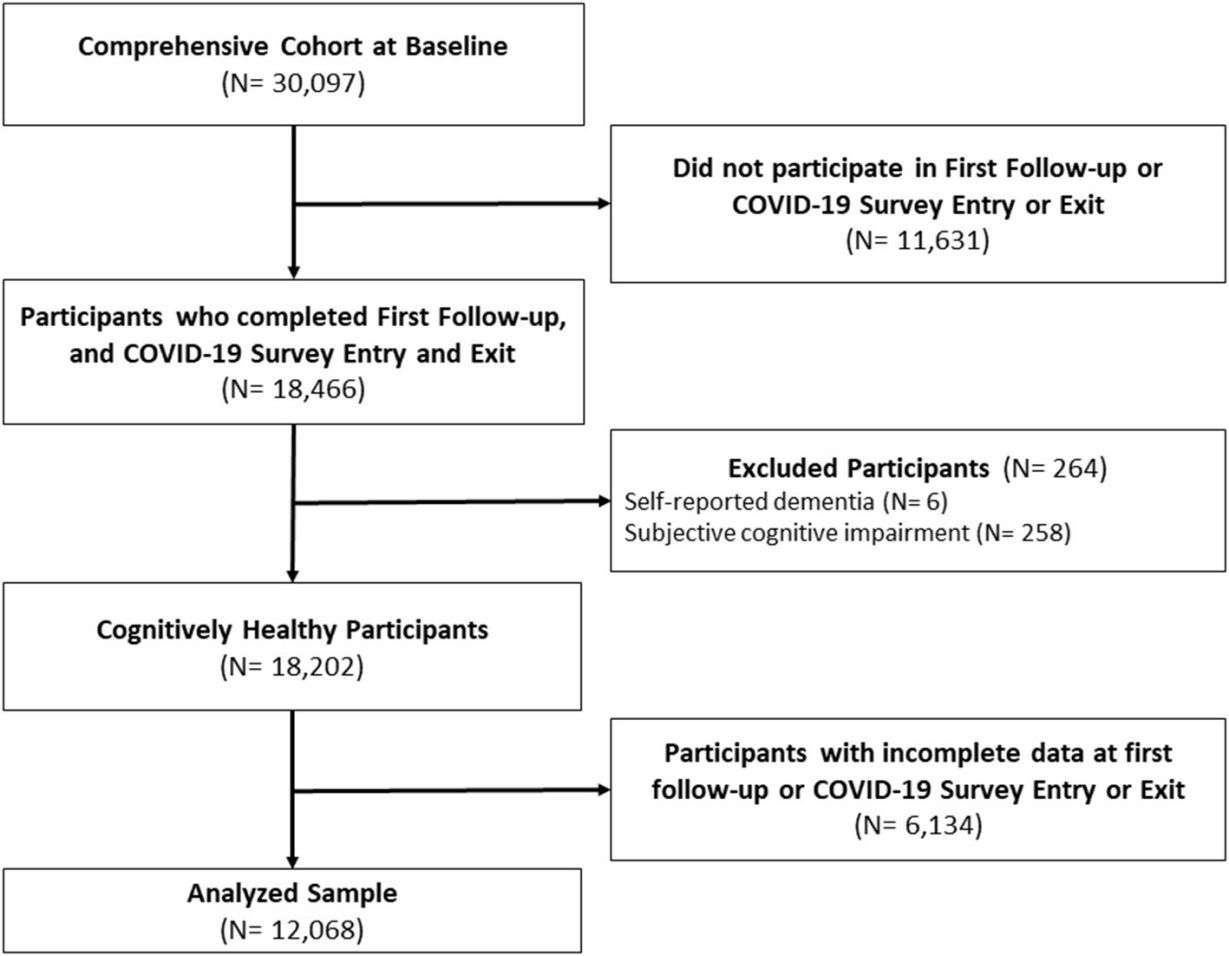
STROBE Diagram. Baseline (2010–2015); First Follow-Up (2015–2018); COVID-19 Survey Entry (April 15–May 30, 2020); COVID-19 Survey Exit (September 29–December 29, 2020).

In this analysis, all participants were from the Comprehensive cohort, cognitively healthy, and provided data at baseline (2010–2015), FU1 (2015–2018), and at COVID-19 entry (April–May 2020) and exit surveys (September–December 2020). We also excluded participants who provided incomplete data at either FU1 or COVID-19 entry or exit surveys. Thus, our final sample size was 12,068. Our STROBE diagram is presented in Figure 1.

### Measures

#### Self-reported mental health disorders

Participants were queried at baseline and FU1 whether they had been diagnosed with a mental health disorder (i.e., depression, anxiety, or a mood disorder). A diagnosis of anxiety was indexed using a single question: “Has a doctor ever told you that you have an anxiety disorder such as a phobia, obsessive-compulsive disorder or a panic disorder?” Depression was characterized by response to the question: “Has a doctor ever told you that you suffer from clinical depression?”. Mood disorders were classified based on response to: “Has a doctor ever told you that you have a mood disorder such as depression (including manic depression), bipolar disorder, mania, or dysthymia?”. Participants who answered “yes” at either baseline or FU1 were classified as having the queried mental health disorder.

#### Pet ownership

Pet ownership was assessed at baseline and FU1 using a single question: “Do you own a pet?”. Participants who responded “yes” were categorized as pet owners; participants who responded “no” or did not respond were classified as non-owners. For our analyses, we indexed pet ownership (yes/no) at FU1, given that these were the most recently available data on pet ownership.

#### 10-Item center for epidemiological studies depression scale

We indexed depressive symptoms using the 10-item Center for Epidemiological Studies Depression Scale (CESD-10); a brief questionnaire for measuring depressive symptoms (19, 20). Scores range from 0 to 30 with higher scores indicating poorer depressive symptoms; a score ≥4 has an 85% positive predictive value for identifying older adults with depression (19). Participants completed the CESD-10 in the home interview at baseline and FU1, and by web or telephone during the COVID-19 Survey. We used continuous CESD-10 scores to examine changes in depressive symptoms from FU1 through COVID-19 exit survey.

#### Generalized anxiety disorder questionnaire

Anxiety symptoms were assessed using the Generalized Anxiety Disorder Questionnaire (GAD-7); a seven item questionnaire for the screening and identification of generalized anxiety disorder symptoms (21). Scores range from 0 to 21 with higher scores indicating greater anxiety symptoms; a score ≥10 has 89% sensitivity and 82% specificity for predicting general anxiety disorder (22). Participants completed the questionnaire either by web or telephone during the COVID-19 Survey. We used the continuous GAD-7 scores to examine anxiety symptoms during the COVID-19 Survey.

#### Covariates

At FU1, participants were queried for age, biological sex, educational attainment, income level, current living status (i.e., house, apartment/condo/town home, assisted living, or other), smoking status (i.e., daily, occasional, or former smoker, or non-smoker), alcohol intake (regular, occasional, or non-drinker), and relationship status (married, divorced/separated, single, or widowed). Body mass index (BMI; kg/m^2^) was indexed using a calibrated scale and stadiometer.

### Statistical analysis

All analyses were conducted in R version 4.0.3 using the lmer package (version 1.1-25). Prior to analysis, all participants with missing data were excluded. Our complete statistical analysis code can be found in a github repository (https://github.com/ryanfalck/CLSA-COVID-19-Project/commit/f5915f7ceb9959ada83ca5c780e41434c1ad1166).

Participant self-reported mental health disorder status was indexed by affirmative responses at either baseline or FU1. For instance, participants who affirmed depression diagnosis at baseline, or participants who affirmed depression diagnosis at FU1, were each indexed as participants with depression. Participants who self-reported more than one mental health disorder at either baseline or FU1 were categorized as having multiple mental health disorders. As a sensitivity analysis, we indexed whether mental health disorder status changed from baseline to FU1. Participants were either classified as having a mental health disorder (1) at baseline, but not FU1; (2) not at baseline, but at FU1; (3) at both baseline and FU1; or (4) neither at baseline or FU1. We conducted separate models for each mental health disorder.

Using data at FU1, we categorized participants as either pet owners, or non-owners based on their response to the question, “Do you own a pet?”. We calculated means, standard deviations and proportions for all variables of interest at baseline.

#### Primary analyses

We used mixed linear models with restricted maximum likelihood estimation to evaluate between-group differences in CESD-10 score during the COVID-19 Survey between people with and without pets based on mental health disorder status. We examined four different mental health disorder statuses: (1) anxiety (i.e., yes/no); (2) depression (yes/no); (3) mood disorder (yes/no); and (4) multiple mental health disorders (multiple vs. no mental health disorders). Time representing COVID-19 Survey assessment (i.e., entry vs. exit) was included as a categorical fixed effect in addition to (1) pet ownership status; (2) mental health disorder status; and (3) the interactions of pet ownership, mental health disorder status, and time. The intercept was specified as a random effect. Each model controlled for CESD-10 score at FU1 in order to account for pre-pandemic depressive symptoms. We thus assumed CESD-10 score at FU1 was the most recent estimate of depressive symptoms prior to COVID-19 entry survey. Additionally, we controlled for age, sex, BMI, income level, educational attainment, living status, smoking status, relationship status, and alcohol intake.

We investigated interaction effects using estimated marginal means, wherein we estimated means and standard errors of CESD-10 score at COVID-19 entry and exit surveys for individuals with and without pets, stratifying for mental health disorder status. We then determined between group differences (i.e., pet owners vs. non-owners) at COVID-19 entry and exit surveys. Subsequently, we examined whether these between group differences (i.e., pet owners—non-owners) were significantly different (*p* < 0.05) between individuals with and without mental health disorders.

#### Secondary analysis

Our secondary analysis examined the interactive associations of pet ownership and mental health disorder status on anxiety symptoms during the COVID-19 Survey. We conducted linear models to examine between-group differences in GAD-7 score at COVID-19 entry and exit surveys based on pet ownership and mental health disorder status (yes/no). Separate analyses were conducted for each mental health disorder (i.e., depression, anxiety, mood disorder, or multiple mental health disorders).

For our models examining GAD-7 score differences at COVID-19 entry survey, we examined the interaction of mental health disorder status and pet ownership; GAD-7 score was the dependent variable of interest. Each model adjusted for the same covariates as our primary models. We conducted similar models examining GAD-7 score differences at COVID-19 exit survey; however, these models also accounted for GAD-7 score differences at COVID-19 entry survey.

Interaction effects were investigated using estimated marginal means of GAD-7 score at COVID-19 entry and exit surveys for individuals with and without pets, stratifying for mental health disorder status. Between group differences at study entry and exit were then calculated, and then between group differences between individuals with and without mental health disorders.

#### Sensitivity analyses

We conducted sensitivity analyses to determine whether changes in mental health status prior to the pandemic affected associations with depressive and anxiety symptoms during the COVID-19 pandemic. Specifically, we examined the interactive associations of pet ownership at FU1 and changes in mental health disorder status from baseline to FU1, and whether this affected CESD-10 scores during the COVID-19 pandemic. Likewise, we conducted a similar sensitivity analysis of the effects on anxiety symptoms during the COVID-19 Survey.

We also conducted a sensitivity analysis to determine if there were sex differences in the interactive associations of pet ownership and mental health disorder status on depressive and anxiety symptoms during the first 9 months of the COVID-19 pandemic. Specifically, we performed stratified analyses for males and females of our primary and secondary analyses in order to determine whether there were sex differences in how pet ownership and mental health disorder status affected CESD-10 scores during the COVID-19 pandemic. We conducted a similar sensitivity analysis of sex differences in the effects on anxiety symptoms during the COVID-19 Survey.

## Results

### Participant characteristics

Table 1 describes participant characteristics of our sample at FU1. Mean age was ~65 years (SD = 9 years), and the sample was 48.9% male. Approximately 81% of participants had a university degree or higher. Participants were suffering from an average of 3.95 (SD = 2.78) chronic conditions, 42.8% owned a pet at baseline; 39.1% owned a pet at FU1. Mean CESD-10 for all participants was 4.45 (SD = 4.20). In total, 17.5% of participants reported a diagnosis of depression at baseline and/or FU1, 9.9% reported anxiety, and 19.7% reported a mood disorder.

**Table 1:** Participant characteristics at first follow-up (2015–2018) for participants (1) in the Comprehensive Cohort with data at first follow-up, and COVID-19 survey entry (April–May 2020) and exit (September–December 2020); and (2) in the analyzed sample.

**Variable**	**Comprehensive cohort (*N* = 18,466)**	**Analytic sample (*N* = 12,068)**
Age	65.22 (9.58)	65.01 (9.24)
Males (*n*, %)	8,864, 48.0%	5,901, 48.9%
Body mass index (kg/m^2^)	28.07 (5.44)	28.04 (5.38)
**Mental health disorder status (***n***, %)**		
Anxiety only	656, 3.7%	403, 3.4%
Depression only	286, 1.6%	192, 1.6%
Mood disorders only	533, 3.0%	340, 2.8%
Multiple mental health disorders	3,136, 17.5%	2,041, 17.0%
No mental health disorders	13,341, 74.5%	9,040, 75.2%
**Education (** ***n*** **, %)**		
Less than high school	752, 4.1%	475, 3.9%
High school diploma	1,575, 8.5%	977, 8.1%
Some university	1,302, 7.1%	825, 6.8%
University degree or higher	14,811, 80.3%	9,791, 81.1%
**Income level (** ***n*** **, %)**		
<$20,000 per year	2,066, 11.7%	1,315, 10.9%
$20,000–$50,000 per year	6,353, 36.0%	4,280, 35.5%
$50,000–$100,000 per year	6,479, 36.7%	4,523, 37.5%
$100,000–$150,000 per year	1,713, 9.7%	1,221, 10.1%
>$150,000 per year	1,056, 6.0%	729, 6.0%
**Living status (** ***n*** **, %)**		
Apartment, condo, or town home	3,441, 18.6%	2,162, 17.9%
Assisted living	165, 0.9%	82, 0.7%
House	14,709, 79.7%	9,740, 80.7%
Other	151, 0.8%	84, 0.7%
Chronic Conditions	4.07 (2.86)	3.95 (2.78)
**Relationship status (** ***n*** **, %)**		
Married	12,940, 70.1%	8,533, 70.7%
Divorced/Separated	2,263, 12.3%	1,447, 12.0%
Single	1,578, 8.6%	1,011, 8.4%
Widowed	1,675, 9.1%	1,077, 8.9%
**Alcohol intake (** ***n*** **, %)**		
Regular drinker	14,295, 77.5%	9,543, 79.1%
Occasional drinker	2,095, 11.4%	1,275, 10.6 %
Non-drinker	2,062, 11.2%	1,250, 10.4%
**Smoking status (** ***n*** **, %)**		
Daily smoker	982, 5.4%	618, 5.1 %
Former smoker	11,132, 60.7%	7,338, 60.8%
Occasional smoker	280, 1.5%	158, 1.3%
Never smoked	5,959, 32.5%	3,954, 32.8%
Pet owner (*n*, %)	7,236, 39.3%	4,718, 39.1%
CESD-10^a^	4.71 (4.34)	4.45 (4.20)
GAD-7^b^	–	–

a 10-Item Center for Epidemiological Studies Depression Scale.

b Generalized Anxiety Disorder Questionnaire (Note: only collected during COVID-19 Survey).

Of the 2,976 participants who reported a mental health disorder at baseline and/or FU1, 68.6% reported having multiple mental health disorders. Tables 2, 3 describes participant characteristics at FU1 stratified by pet ownership and mental health disorder status, respectively.

**Table 2:** Analyzed sample participant characteristics at first follow-up (2015–2018) based on pet ownership.

**Variable**	**Pet owners (*N =* 4,718)**	**Non-owners (*N =* 7,350)**
Age	62.03 (8.52)	67.02 (9.21)
Males (*n*, %)	2,139, 45.3%	3,920, 50.8%
BMI (kg/m^2^)	28.37 (5.66)	27.85 (5.19)
**Mental health disorder status (** ***n*** **, %)**		
Anxiety only	180, 3.8%	223, 3.0%
Depression only	81, 1.7%	111, 1.5%
Mood disorders only	150, 3.2%	190, 2.6%
Multiple mental health disorders	964, 20.5%	1,077, 14.7%
No mental health disorders	3,319, 70.7%	5,721, 78.1%
**Education (** ***n*** **, %)**		
Less than a high school diploma	145, 3.1%	330, 4.5%
High school diploma	360, 7.6%	617, 8.4%
Some university	339, 7.2%	486, 6.6%
University degree or higher	3,874, 82.1%	5,917, 80.5%
**Income level (** ***n*** **, %)**		
<$20,000 per year	480, 10.2%	835, 11.4%
$20,000–$50,000 per year	1,546, 32.8%	2,734, 37.2%
$50,000–$100,000 per year	1,799, 38.1%	2,724, 37.1%
$100,000–$150,000 per year	535, 11.3%	686, 9.3%
>$150,000 per year	358, 7.6%	371, 5.0%
**Living status (** ***n*** **, %)**		
Apartment, condo, or town home	447, 9.5%	1,715, 23.3%
Assisted living	15, 0.3%	67, 0.9%
House	4,225, 89.6%	5,515, 75.0%
Other	31, 0.7%	53, 0.7%
Chronic conditions	3.84 (2.76)	4.02 (2.78)
**Relationship status (** ***n*** **, %)**		
Married	3,524, 74.7%	5,009, 68.1%
Divorced/Separated	539, 11.5%	908, 12.3%
Single	328, 7.0%	683, 9.3%
Widowed	327, 6.9%	750, 10.2%
**Alcohol intake (** ***n*** **, %)**		
Regular drinker	3,716, 78.8%	5,827, 79.3%
Occasional drinker	543, 11.5%	732, 10.0%
Non-drinker	459, 9.7%	791, 10.8%
**Smoking status (** ***n*** **, %)**		
Daily smoker	287, 6.1%	331, 4.5%
Former smoker	2,850, 60.4%	4,488, 61.1%
Occasional smoker	73, 1.2%	85, 1.2%
Never smoked	1,508 (32.0%)	2,446, 33.3%
CESD-10^a^	3.84 (2.76)	4.02 (2.78)
GAD-7^b^	–	–

a 10-Item Center for Epidemiological Studies Depression Scale.

b Generalized Anxiety Disorder Questionnaire (Note: only collected during COVID-19 Survey).

**Table 3:** Analyzed sample participant characteristics at first follow-up (2015–2018) by self-reported mental health disorder status.

**Variable**	**Anxiety (*N =* 403)**	**Depression (*N =* 192)**	**Mood disorders (*N =* 340)**	**Multiple mental health disorders (*N =* 2,041)**	**No mental health disorders (*N =* 9,040)**
Age	63.57 (8.59)	64.10 (8.47)	64.01 (9.08)	63.30 (8.28)	65.51 (9.44)
Males (*n*, %)	171, 42.4%	66, 34.4%	130, 38.2%	730, 35.8%	4,782, 52.9%
BMI (kg/m^2^)	28.03 (6.06)	28.48 (5.92)	28.68 (6.43)	29.28 (6.03)	27.72 (5.09)
**Education (** ***n*** **, %)**					
Less than a high school diploma	20, 5.0%	11, 5.7%	15,4.4%	84, 4.1%	344, 3.8%
High school diploma	32, 7.9%	15, 7.8%	35, 10.3%	152, 7.4%	735, 8.1%
Some university	37, 9.2%	9, 4.7%	38, 11.2%	171, 8.4%	567, 6.3%
University degree or higher	314, 77.9%	157, 81.8%	252, 74.1%	1,634, 80.1%	7,394, 81.8%
**Income level (** ***n*** **, %)**					
< $20,000 per year	61, 15.1%	28, 14.6%	47, 13.8%	333, 16.3%	838, 9.3%
$20,000–$50,000 per year	151, 37.5%	85, 44.3%	131, 38.5%	786, 38.5%	3,108, 34.4%
$50,000–$100,000 per year	134, 33.3%	59, 30.7%	113, 33.2%	692, 33.9%	3,508, 38.8%
$100,000–$150,000 per year	34, 8.4%	14, 7.3%	34, 10.0%	162, 7.9%	838, 9.3%
>$150,000 per year	23, 5.7%	6, 3.1%	15, 4.4%	68, 3.3%	617, 6.8%
**Living status (** ***n*** **, %)**					
Apartment, condo, or town home	69, 17.1%	39, 20.3%	71, 20.9%	472, 23.1%	1,498, 16.6%
Assisted living	1, 0.2%	4, 2.1%	4, 1.2%	16, 0.8%	57, 0.6%
House	330, 81.9%	149, 77.6%	261, 76.8%	1,532, 75.1%	7,429, 82.2%
Other	3, 0.7%	0, 0.0%	4, 1.2%	21, 1.0%	56, 0.6%
Chronic conditions	4.57 (3.04)	4.57 (2.96)	4.75 (3.04)	4.99 (2.97)	3.65 (2.64)
**Relationship status (** ***n*** **, %)**					
Married	293, 72.7%	108, 56.2%	213, 62.6%	1,250, 61.2%	6,640, 73.5%
Divorced/Separated	44, 10.9%	39, 20.3%	55, 16.2%	395, 19.3%	905, 10.1%
Single	34, 8.4%	24, 12.5%	38, 11.2%	245, 12.5%	661, 7.3%
Widowed	32, 7.9%	21, 10.9%	34, 10.0%	151, 7.4%	834, 9.2%
**Alcohol intake (** ***n*** **, %)**					
Regular drinker	302, 74.9%	149, 77.6%	256, 75.3%	1,492, 73.1%	7,308, 80.8%
Occasional drinker	46, 11.4%	21, 10.9%	49, 14.4%	292, 14.3%	859, 9.5%
Non-drinker	55, 13.6%	22, 11.5%	35, 10.3%	257,12.6%	873, 9.7%
**Smoking status (** ***n*** **, %)**					
Daily smoker	28, 6.9%	21, 10.9%	21, 6.2%	164, 8.0%	380, 4.2%
Former smoker	248, 61.5%	115, 59.9%	221, 65.0%	1,220, 59.8%	5,501, 60.9%
Occasional smoker	7, 1.7%	4, 2.1%	7, 2.1%	31, 1.5%	109, 1.2%
Never smoked	120, 29.8%	52, 27.1%	91, 26.8%	626, 30.7%	3,050, 33.7%
Pet owner (*n*, %)	180, 44.7%	81, 42.2%	150, 44.1%	964, 47.2%	3,440, 36.5%
CESD-10^a^	5.52 (4.63)	4.95 (3.95)	6.40 (5.16)	7.05 (5.62)	3.71 (3.42)
GAD-7^b^	–	–	–	–	–

a 10-Item Center for Epidemiological Studies Depression Scale.

b Generalized Anxiety Disorder Questionnaire (Note: only collected during COVID-19 Survey.

### Differences in the associations of pet ownership with CESD-10 score during COVID-19 based on mental health disorder status

Our models examining the associations of pet ownership with CESD-10 score based on mental health disorder status are described in Table 4. Compared with participants without mental health disorders, those with mental health disorders (i.e., anxiety, depression, mood disorders, or multiple mental health disorders) reported higher CESD-10 score at FU1, and COVID-19 entry and exit surveys (*p* < 0.001).

**Table 4:** Estimated marginal means and standard errors for changes in 10-item Center for Epidemiological Studies Depression Scale (CESD-10) score at first follow-up ([FU1]; 2015–2018), and COVID-19 Survey entry (April 15–May 30, 2020) and exit (September 29–December 29, 2020), based on self-reported mental health disorder status and pet ownership.

	**Pet owners**			**Non-pet owners**			**Between group differences (Pet owners—Non-pet owners)**			
	**FU1 mean (SD)**	**COVID-19 survey entry**	**COVID-19 survey exit**	**FU1 mean (SD)**	**COVID-19 survey entry**	**COVID-19 survey exit**	**Estimated difference at COVID-19 survey entry (95% CI)**	***p***	**Estimated difference at COVID-19 survey exit (95% CI)**	***p***
***Anxiety***											
Anxiety (*N =* 1,197)	7.37 (5.98)	6.89 ± 0.26	7.68 ± 0.26	7.23 (5.74)	6.26 ± 0.25	6.67 ± 0.25	0.63 (0.14, 1.12)	0.012	1.02 ^β^ (0.53, 1.51)	<0.001
No anxiety (*N =* 10,862)	4.27 (3.95)	5.66 ± 0.19	5.85 ± 0.19	4.05 (3.79)	5.45 ± 0.19	5.62 ± 0.19	0.22 (0.04, 0.39)	0.015	0.23 (0.06, 0.41)	0.008
***Depression***											
Depression (*N =* 2,109)	7.08 (5.71)	7.08 ± 0.23	7.67 ± 0.23	6.64 (5.33)	6.41 ± 0.22	6.71 ± 0.22	0.67 ^α^ (0.30, 1.03)	0.003	0.96 ^β^ (0.59, 1.33)	< 0.001
No depression (*N =* 9,911)	3.99 (3.66)	5.44 ± 0.19	5.61 ± 0.19	3.89 (3.65)	5.31 ± 0.19	5.48 ± 0.19	0.13 (−0.05, 0.31)	0.155	0.13 (−0.05, 0.31)	0.168
***Mood disorders***											
Mood disorders (*N =* 2,369)	7.13 (5.68)	7.03 ± 0.22	7.77 ± 0.22	6.83 (5.49)	6.47 ± 0.21	6.79 ± 0.21	0.56 ^α^ (0.21, 0.91)	0.002	0.98 ^β^ (0.63, 1.33)	< 0.001
No mood disorders (*N =* 9,685)	3.89 (3.54)	5.38 ± 0.19	5.50 ± 0.19	3.79 (3.51)	5.26 ± 0.19	5.43 ± 0.19	0.13 (−0.06, 0.31)	0.174	0.08 (−0.11, 0.26)	0.396
***Multiple mental health disorders***										
Multiple disorders (*N =* 2,041)	7.24 (5.74)	7.21 ± 0.23	7.89 ± 0.23	6.89 (5.50)	6.42 ± 0.22	6.84 ± 0.22	0.79 ^α^ (0.42, 1.16)	< 0.001	1.05 ^β^ (0.68, 1.42)	< 0.001
No mental health disorders (*N =* 9,040)	3.77 (3.41)	5.31 ± 0.19	5.41 ± 0.19	3.69 (3.42)	5.19 ± 0.19	5.37 ± 0.19	0.12 (−0.07, 0.31)	0.211	0.05 (−0.14, 0.23)	0.636

All models controlling for age, sex, BMI, income level, educational attainment, living status, smoking status, relationship status, alcohol intake, and CESD-10 score at FU1.

α Significantly different (*p* < 0.05) between group difference between individuals with and without mental health disorders at COVID-19 Survey entry.

β Significantly different (*p* < 0.05) between group difference between individuals with and without mental health disorders at COVID-19 Survey exit.

Among those without anxiety, participants with pets had higher CESD-10 scores at both COVID-19 entry (estimated mean difference [EMD]: 0.22; 95% CI: [0.04, 0.39]; *p* = 0.015) and exit surveys (EMD: 0.23; 95% CI: [0.06, 0.41]; *p* = 0.008) as compared with the same sub-population without pets. Individuals without depression who owned pets did not significantly differ in CESD-10 score at either COVID-19 entry and exit surveys as compared to individuals without depression who were not pet owners. Similarly, there were no significant differences in CESD-10 score at either COVID-19 entry or exit surveys between individuals without mood disorders who were pet owners and their peers without pets.

Participants with either anxiety, depression, or a mood disorder and pets had higher CESD-10 scores at COVID-19 entry (EMD range: 0.56–0.67; *p* < 0.05) and exit survey (EMD range: 0.96–1.02; *p* < 0.05) compared to their peers with mental health disorders and no pets. Participants with anxiety had a significantly greater difference in CESD-10 score between those who owned pets and non-pet owners at COVID-19 exit survey (EMD: 0.78; 95% CI: [0.27, 1.29]; *p* = 0.003) as compared to those same sub-populations for people without mental health disorders. Participants with either depression or a mood disorder who owned pets had a significantly greater difference in CESD-10 score at COVID-19 entry (EMD range: 0.50–0.54; *p* < 0.05) and exit surveys (EMD range: 0.83–0.90; *p* < 0.05) compared to those same sub-populations for people without mental health disorders.

Participants with multiple mental health disorders and pets had higher CESD-10 scores at COVID-19 entry (EMD: 0.79; 95% CI: [0.42, 1.16]; *p* < 0.001) and exit surveys (EMD: 1.05; 95% CI: [0.68, 1.42]; *p* < 0.001) than their non-owner peers. Individuals with multiple mental health disorders who owned pets had significantly greater differences in CESD-10 scores at COVID-19 entry (EMD: 0.67; 95% CI: [0.26, 1.08]; *p* = 0.002) and exit surveys (EMD: 1.15; 95% CI: [0.52, 1.78]; *p* < 0.001) compared to those same sub-populations for people without mental health disorders.

### Differences in the association of pet ownership with anxiety symptoms during COVID-19 based on mental health disorder status

We describe the results of our models examining the associations of pet ownership with anxiety symptoms based on mental health disorder status in Table 5. Compared with participants without a mental health disorder, participants with mental health disorders reported higher GAD-7 score at both COVID-19 entry and exit surveys (*p* < 0.001).

**Table 5:** Estimated marginal means and standard errors for General Anxiety Disorder Questionnaire (GAD-7) score at COVID-19 Survey entry (April 15–May 30, 2020) and exit (September 29–December 29, 2020), based on mental health disorder status and pet ownership.

	**Pet owners**		**Non-pet owners**		**Between group differences (Pet owners—Non-pet owners)**			
	**COVID-19 survey entry**	COVID-19 survey exit^*^	**COVID-19 survey entry**	COVID-19 survey exit^*^	**Estimated difference at COIVD-19 survey entry (95% CI)**	***p***	Estimated difference at COIVD-19 survey exit (95% CI)^*^	***p***
***Anxiety***									
Anxiety (*N =* 1,197)	4.45 ± 0.23	3.71 ± 0.17	4.02 ± 0.22	3.14 ± 0.16	0.44 (0.06, 0.82)	0.023	0.57 ^β^ (0.27, 0.87)	< 0.001
No anxiety (*N =* 10,862)	2.48 ± 0.19	2.39 ± 0.13	2.32 ± 0.18	2.26 ± 0.13	0.17 (0.03, 0.30)	0.014	0.13 (0.02, 0.23)	0.019
***Depression***								
Depression (*N =* 2,109)	4.43 ± 0.19	3.28 ± 0.15	3.99 ± 0.18	2.89 ± 0.15	0.43 (0.15, 0.72)	0.003	0.39 ^β^ (0.17, 0.62)	< 0.001
No depression (*N =* 9,911)	2.78 ± 0.17	2.34 ± 0.13	2.65 ± 0.16	2.23 ± 0.13	0.13 (−0.01, 0.27)	0.071	0.11 (−0.01, 0.22)	0.051
***Mood disorders***								
Mood disorders (*N =* 2,369)	4.41 ± 0.19	3.30 ± 0.15	4.14 ± 0.18	2.92 ± 0.14	0.28 (0.01, 0.55)	0.043	0.37 ^β^ (0.16, 0.59)	< 0.001
No mood disorders (*N =* 9,685)	2.69 ± 0.17	2.29 ± 0.13	2.55 ± 0.16	2.18 ± 0.13	0.13 (−0.01, 0.27)	0.064	0.10 (−0.01, 0.22)	0.069
***Multiple mental health disorders***								
Multiple disorders (*N =* 2,041)	4.50 ± 0.19	3.42 ± 0.15	4.10 ± 0.18	2.96 ± 0.15	0.40 (0.11, 0.69)	0.007	0.46 (0.23, 0.69)	< 0.001
No mental health disorders (*N =* 9,040)	2.55 ± 0.17	2.23 ± 0.13	2.46 ± 0.16	2.12 ± 0.13	0.10 (−0.05, 0.24)	0.199	0.10 ^β^ (0.01, 0.22)	0.081

All models controlling for age, sex, BMI, income level, educational attainment, living status, smoking status, relationship status, and alcohol intake.

* Model also accounts for GAD-7 score at COVID-19 Survey entry.

^α^Significantly different (*p* < 0.05) between group difference between individuals with and without mental health disorders at COVID-19 Survey entry.

β Significantly different (*p* < 0.05) between group difference between individuals with and without mental health disorders at COVID-19 Survey entry.

Among those without anxiety, participants with pets had higher GAD-7 scores at both COVID-19 entry (EMD: 0.17; 95% CI: [0.043 0.30]; *p* = 0.014) and exit surveys (EMD: 0.13; 95% CI: [0.02, 0.23]; *p* = 0.019) compared to participants without pets. Individuals without depression who owned pets did not significantly differ in GAD-7 score at either COVID-19 entry and exit surveys as compared to individuals without depression who were not pet owners. Similarly, there were no significant differences in GAD-7 score at either COVID-19 entry or exit surveys between individuals without mood disorders who were pet owners and their peers without pets.

Participants with either anxiety, depression, or a mood disorder and pets had higher GAD-7 scores at COVID-19 entry (EMD range: 0.28–0.44; *p* < 0.05) and exit surveys (EMD range: 0.37–0.57; *p* < 0.05) compared to their peers with mental health disorders and no pets. Participants with anxiety, depression, or a mood disorder and pets had a significantly greater difference in GAD-7 score between pet owners and non-owners at COVID-19 exit survey (EMD range: 0.27–0.44; *p* < 0.05) compared to people without mental health disorders.

Participants with multiple mental health disorders and pets had higher GAD-7 scores at COVID-19 entry (EMD: 0.40; 95% CI: [0.11, 0.69]; *p* = 0.007) and exit surveys (EMD: 0.46; 95% CI: [0.23, 0.69]; *p* < 0.001) than their non-owner peers. We also found that participants with multiple mental health disorders and pets had a significantly greater difference in GAD-7 score between pet owners and non-owners at COVID-19 exit survey (EMD: 0.35; 95% CI: [0.10, 0.60]; *p* = 0.007) compared to people without mental health disorders.

### Sensitivity analyses

Table 6 describes differences in CESD-10 scores at COVID-19 entry and exit surveys based on changes in mental health disorder status from baseline to first-follow-up. Pet owners who self-reported being diagnosed with a mental health disorder at either baseline, FU1, or both time points, had higher CESD-10 scores at either COVID-19 entry or exit surveys compared with non-owners. Participants who did not report having a mental health disorder at any time point and were pet owners did not have different CESD-10 scores from non-owners at either COVID-19 entry or exit surveys.

**Table 6:** Estimated marginal means and standard errors for changes in 10-item Center for Epidemiological Studies Depression Scale (CESD-10) score at FU1 (i.e., 2015–2018), and COVID-19 Survey entry and exit based on changes in mental health disorder status from baseline (i.e., 2010–2015) to FU1 and pet ownership at FU1.

	**Pet owners**			**Non-pet owners**			**Between group differences**			
	**FU1 mean (SD)**	**COVID-19 survey entry**	**COVID-19 survey exit**	**FU1 mean (SD)**	**COVID-19 survey entry**	**COVID-19 survey exit**	**Estimated difference at COVID-19 survey entry (95% CI)**	***p***	**Estimated difference at COVID-19 survey exit (95% CI)**	***p***
***Anxiety***										
Baseline only (*N =* 145)	5.44 (4.90)	6.77± 0.57	7.54 ± 0.57	6.03 (4.92)	5.73 ± 0.51	5.66 ± 0.51	1.04 (−0.38, 2.45)	0.151	1.89 (0.47, 3.30)	0.009
FU1 only (*N =* 308)	7.89 (5.87)	6.14 ± 0.40	7.61 ± 0.40	6.79 (5.85)	6.39 ± 0.39	6.60 ± 0.39	−0.25 (−0.71, 1.21)	0.613	1.01 (0.04, 1.97)	0.040
Baseline and first-follow-up (*N =* 744)	8.68 (6.60)	7.22 ± 0.29	7.74 ± 0.29	8.34 (7.07)	6.31 ± 0.28	6.90 ± 0.28	0.91 (0.29, 1.54)	0.004	0.84 (0.22, 1.46)	0.008
No anxiety (*N =* 10,862)	4.31 (3.97)	5.66 ± 0.19	5.85 ± 0.19	4.10 (3.81)	5.44 ± 0.19	5.61 ± 0.19	0.22 (0.04, 0.39)	0.015	0.23 (0.06, 0.41)	0.008
***Depression***										
Baseline only (*N =* 406)	6.71 (5.73)	6.86 ± 0.37	7.25 ± 0.37	5.62 (4.66)	6.63 ± 0.33	6.37 ± 0.33	0.22 (−0.62, 1.06)	0.603	0.87 (−0.03, 1.71)	0.042
FU1 only (*N =* 287)	6.88 (5.34)	6.28 ± 0.45	7.14 ± 0.45	7.74 (5.84)	6.42 ± 0.37	6.85 ± 0.37	−0.14 (−0.89, 1.16)	0.793	0.29 (−0.74, 1.31)	0.584
Baseline and first-follow-up (*N =* 1,416)	7.21 (5.77)	7.26 ± 0.24	7.87 ± 0.24	6.69 (5.35)	6.34 ± 0.24	6.79 ± 0.24	0.92 (0.47, 1.36)	< 0.001	1.07 (0.63, 1.52)	< 0.001
No depression (*N =* 9,911)	4.04 (3.69)	5.44 ± 0.19	5.61 ± 0.19	3.93 (3.65)	5.31 ± 0.19	5.49 ± 0.19	0.13 (−0.05, 0.31)	0.154	0.13 (−0.05, 0.31)	0.167
***Mood disorders***										
Baseline only (*N =* 9,685)	6.48 (5.14)	6.92 ± 0.46	8.13 ± 0.46	6.47 (5.12)	6.66 ± 0.41	6.78 ± 0.41	0.26 (−0.84, 1.37)	0.640	1.35 (0.24, 2.46)	0.017
FU1 only (*N =* 455)	7.00 (5.37)	6.06 ± 0.36	6.83 ± 0.36	7.05 (5.66)	5.98 ± 0.32	6.43 ± 0.32	−0.09 (−0.71, 0.89)	0.829	0.40 (−0.40, 1.20)	0.325
Baseline and first-follow-up (*N =* 1,681)	7.24 (5.81)	7.29 ± 0.23	7.95 ± 0.23	6.81 (5.49)	6.62 ± 0.23	6.92 ± 0.23	0.67 (0.26, 1.08)	0.002	1.03 (0.62, 1.44)	< 0.001
No mood disorders (*N =* 9,685)	3.94 (3.57)	5.39 ± 0.19	5.51 ± 0.19	3.84 (3.52)	5.27 ± 0.19	5.44 ± 0.19	0.12 (−0.06, 0.31)	0.179	0.07 (−0.11, 0.26)	0.421

All models controlling for age, sex, BMI, income level, educational attainment, living status, smoking status, relationship status, alcohol intake, and CESD-10 score at FU1.

Table 7 describes differences in GAD-7 scores at COVID-19 entry and exit surveys based on 1) changes in mental health disorder status from baseline to first-follow-up and 2) pet ownership at FU1. Participants who reported having a mental health disorder at either baseline, FU1, or both time points and were pet owners had higher GAD-7 scores at either COVID-19 entry or exit surveys compared with non-owners. Participants who did not report having a mental health disorder at any time point and were pet owners did not have different GAD-7 scores from non-owners at either COVID-19 entry or exit surveys.

**Table 7:** Estimated marginal means and standard errors for changes in General Anxiety Disorder Questionnaire (GAD-7) score at COVID-19 Survey entry and exit based on changes in mental health disorder status from baseline (i.e., 2010–2015) to FU1 (2015–2018) and pet ownership at FU1.

	**Pet owners**		**Non-Pet owners**		**Between group differences**			
	**COVID-19 survey entry**	COVID-19 survey exit^*^	**COVID-19 survey entry**	COVID-19 survey exit^*^	**Estimated difference at COVID-19 survey entry (95% CI)**	***p***	Estimated difference at COVID-19 survey exit (95% CI)^*^	***p***
***Anxiety***								
Baseline only (*N =* 145)	4.06 ± 0.45	4.27 ± 0.44	2.95 ± 0.40	2.91 ± 0.39	1.11 (0.02, 2.20)	0.046	1.36 (0.29, 2.43)	0.013
FU1 only (*N =* 308)	4.98 ± 0.31	5.17 ± 0.31	4.13 ± 0.31	4.00 ± 0.30	0.85 (0.10, 1.59)	0.026	1.17 (0.09, 1.04)	0.002
Baseline and first-follow-up (*N =* 744)	5.03 ± 0.23	5.25 ± 0.23	4.91 ± 0.23	4.69 ± 0.23	0.13 (−0.35, 0.61)	0.605	0.56 (0.44, 1.90)	0.019
No anxiety (*N =* 10,862)	2.90 ± 0.17	2.62 ± 0.16	2.73 ± 0.16	2.40 ± 0.16	0.17 (0.03, 0.30)	0.015	0.23 (0.09, 0.36)	0.001
***Depression***								
Baseline only (*N =* 406)	4.50 ± 0.30	4.58 ± 0.29	3.72 ± 0.27	3.25 ± 0.27	0.78 (−0.13, 1.43)	0.019	1.33 (0.69, 1.97)	< 0.001
FU1 only (*N =* 287)	3.83 ± 0.36	4.21 ± 0.29	4.51 ± 0.29	3.47 ± 0.35	−0.67 (−1.47, 0.12)	0.097	−0.74 (−1.52, 0.04)	0.064
Baseline and first-follow-up (*N =* 1,416)	4.49 ± 0.20	4.52 ± 0.20	3.95 ± 0.20	3.82 ± 0.20	0.55 (0.20, 0.90)	0.002	0.70 (0.35, 1.04)	< 0.001
No depression (*N =* 9,911)	2.77 ± 0.17	2.50 ± 0.17	2.64 ± 0.16	2.31 ± 0.16	0.13 (−0.01, 0.27)	0.072	0.19 (0.05, 0.33)	0.001
***Mood disorders***								
Baseline only (*N =* 9,685)	4.85 ± 0.37	4.69 ± 0.36	3.67 ± 0.32	3.57 ± 0.32	1.18 (0.32, 2.04)	0.007	1.12 (0.27, 1.97)	0.010
FU1 only (*N =* 455)	3.85 ± 0.29	3.74 ± 0.28	4.15 ± 0.25	3.93 ± 0.25	−0.31 (−0.93, 0.31)	0.334	0.18 (−0.79, 0.43)	0.557
Baseline and first-follow-up (*N =* 1,681)	4.50 ± 0.20	4.55 ± 0.19	4.22 ± 0.19	3.93 ± 0.19	0.29 (−0.03, 0.60)	0.079	0.62 (0.31, 0.93)	< 0.001
No mood disorders (*N =* 9,685)	2.70 ± 0.17	2.41 ± 0.17	2.56 ± 0.16	2.23 ± 0.16	0.13 (−0.01, 0.28)	0.066	0.18 (0.04, 0.32)	0.011

All models controlling for age, sex, BMI, income level, educational attainment, living status, smoking status, relationship status, and alcohol intake;

* Model also accounts for GAD-7 score at COVID-19 Survey entry.

Table 8 describes sex differences in CESD-10 scores at COVID-19 entry and exit surveys based on mental health disorder status and pet ownership at first-follow-up. Females had higher CESD-10 scores than males for both individuals with and without mental health disorders. Females with anxiety and pets had a smaller difference in CESD-10 score from females without pets and anxiety at COVID-19 exit survey compared to their male peers. All other results were similar between males and females and mirrored the results of our primary analysis.

**Table 8:** Estimated marginal means and standard errors for sex differences in changes in 10-item Center for Epidemiological Studies Depression Scale (CESD-10) score at FU1 (i.e., 2015–2018), and COVID-19 Survey entry and exit based on mental health disorder status and pet ownership.

	**Pet owners**			**Non-pet owners**			**Between group differences (Pet owners—Non-pet owners)**			
	**FU1 mean (SD)**	**COVID-19 survey entry**	**COVID-19 survey exit**	**FU1 mean (SD)**	**COVID-19 survey entry**	**COVID-19 survey exit**	**Estimated difference at COVID-19 survey entry (95% CI)**	***p***	**Estimated difference at COVID-19 survey exit (95% CI)**	***p***
***Anxiety***										
**Males**										
Anxiety (*N =* 429)	6.61 (5.52)	5.76 ± 0.37	7.08 ± 0.37	6.85 (5.77)	4.95 ± 0.36	5.55 ± 0.36	0.80 (0.07, 1.53)	0.031	1.53 (0.81, 2.26)	< 0.001
No anxiety (*N =* 5,469)	3.71 (3.51)	4.43 ± 0.27	4.74 ± 0.27	3.73 (3.52)	4.38 ± 0.26	4.72 ± 0.26	0.05 (−0.17, 0.27)	0.627	0.03 (−0.19, 0.24)	0.823
**Females**										
Anxiety (*N =* 768)	7.77 (6.16)	7.98 ± 0.36	7.68 ± 0.26	7.45 (5.71)	8.51 ± 0.36	7.77 ± 0.36	0.51 (−0.16, 1.18)	0.133	0.74^**^ (0.07, 1.41)	0.031
No anxiety (*N =* 5,393)	4.77 (4.24)	6.89 ± 0.28	6.52 ± 0.28	4.40 (4.03)	6.97 ± 0.28	6.51 ± 0.28	0.36 (0.10, 0.63)	0.015	0.46 (0.20, 0.73)	< 0.001
***Depression***										
**Males**										
Depression (*N =* 758)	6.36 (5.31)	5.77 ± 0.33	6.65 ± 0.33	6.41 (5.30)	5.36 ± 0.31	5.80 ± 0.31	0.42 (−0.14, 0.97)	0.140	0.86 (0.31, 1.41)	0.002
No depression (*N =* 5,123)	3.55 (3.33)	4.28 ± 0.27	4.59 ± 0.27	3.58 (3.37)	4.23 ± 0.26	4.58 ± 0.26	0.05 (−0.18, 0.27)	0.675	0.02 (−0.21, 0.24)	0.879
**Females**										
Depression (*N =* 1,351)	7.43 (5.87)	8.25 ± 0.32	8.71 ± 0.32	6.79 (5.35)	7.43 ± 0.32	7.65 ± 0.32	0.82 (0.31, 1.32)	0.002	1.06 (0.56, 1.56)	< 0.001
No depression (*N =* 4,788)	4.41 (3.91)	6.60 ± 0.28	6.64 ± 0.28	4.24 (3.92)	6.40 ± 0.28	6.38 ± 0.28	0.20 (−0.08, 0.48)	0.154	0.26 (−0.02, 0.54)	0.072
***Mood disorders***										
**Males**										
Mood disorders (*N =* 864)	6.42 (5.26)	5.77 ± 0.32	6.76 ± 0.32	6.51 (5.49)	5.30 ± 0.30	5.73 ± 0.30	0.47 (−0.05, 0.99)	0.074	1.04 (0.52, 1.55)	< 0.001
No mood disorders (*N =* 5,031)	3.48 (3.25)	4.26 ± 0.27	4.54 ± 0.27	3.51 (3.25)	4.24 ± 0.26	4.59 ± 0.26	0.02 (−0.21, 0.25)	0.852	−0.05 (−0.28, 0.18)	0.679
**Females**										
Mood disorders (*N =* 1,505)	7.48 (5.48)	8.17 ± 0.31	8.77 ± 0.31	7.04 (5.48)	7.56 ± 0.31	7.79 ± 0.31	0.61 (0.13, 1.08)	0.012	0.98 (0.50, 1.45)	< 0.001
No mood disorders (*N =* 4,654)	4.28 (3.76)	6.50 ± 0.29	6.47 ± 0.29	4.11 (3.76)	6.27 ± 0.28	6.23 ± 0.28	0.23 (−0.05, 0.51)	0.114	0.23 (−0.05, 0.52)	0.111

All models controlling for age, sex, BMI, income level, educational attainment, living status, smoking status, relationship status, alcohol intake, and CESD-10 score at FU1.

** Significant (*p* < 0.05) difference between males and females at COVID-19 Survey exit.

Table 9 describes sex differences in GAD-7 scores at COVID-19 entry and exit survey based on mental health disorder status and pet ownership at first-follow-up. Females had higher GAD-7 scores than males for both those with and without mental health disorders. Females without anxiety who were pet owners had a larger difference in GAD-7 score from females without anxiety who were not owners at COVID-19 entry and exit surveys compared to their male peers. Females with depression who were pet owners had a larger difference in GAD-7 score from females with depression who were not owners at COVID-19 entry and exit surveys compared to their male peers. All other results were similar between males and females and mirrored the results of our secondary analysis.

**Table 9:** Estimated marginal means and standard errors for sex differences in General Anxiety Disorder Questionnaire (GAD-7) score at COVID-19 Survey entry and exit, based on mental health disorder status and pet ownership.

	**Pet owners**		**Non-pet owners**		**Between group differences (Pet owners—Non-pet owners)**			
	**COVID-19 survey entry**	COVID-19 survey exit^*^	**COVID-19 survey entry**	COVID-19 survey exit^*^	**Estimated difference at COIVD-19 survey entry (95% CI)**	***p***	Estimated difference at COIVD-19 survey exit (95% CI)^*^	***p***
***Anxiety***										
**Males**										
Anxiety (*N =* 429)	4.57 ± 0.31	4.73 ± 0.31	3.94 ± 0.29	3.74 ± 0.30	0.63 (0.06, 1.20)	0.030	0.99 (0.42, 1.56)	<0.001
No anxiety (*N =* 5,469)	2.12 ± 0.23	1.84 ± 0.23	2.15 ± 0.23	1.75 ± 0.23	−0.03 (−0.20, 0.14)	0.736	0.09 (−0.09, 0.26)	0.328
**Females**										
Anxiety (*N =* 768)	5.36 ± 0.30	5.62 ± 0.29	4.99 ± 0.30	4.87 ± 0.29	0.37 (−0.15, 0.88)	0.166	0.75 (0.25, 1.25)	< 0.001
No anxiety (*N =* 5,393)	3.65 ± 0.25	3.39 ± 0.24	3.28 ± 0.24	3.03 ± 0.24	0.37 ^α^ (0.17, 0.58)	< 0.001	0.37 ^β^ (0.17, 0.57)	0.019
***Depression***										
**Males**										
Depression (*N =* 758)	3.55 ± 0.27	3.28 ± 0.15	3.59 ± 0.28	3.14 ± 0.26	0.05 (−0.38, 0.48)	0.812	0.46 (0.02, 0.89)	0.039
No depression (*N =* 5,123)	2.07 ± 0.23	2.34 ± 0.13	1.76 ± 0.23	1.64 ± 0.23	0.04 (−0.14, 0.22)	0.672	0.12 (−0.06, 0.30)	0.178
**Females**										
Depression (*N =* 1,351)	5.14 ± 0.27	5.13 ± 0.26	4.48 ± 0.27	4.36 ± 0.26	0.66 ^α^ (0.27, 1.06)	< 0.001	0.77 ^β^ (0.39, 1.15)	< 0.001
No depression (*N =* 4,788)	3.43 ± 0.25	3.20 ± 0.24	3.19 ± 0.25	2.94 ± 0.24	0.24 (0.02, 0.46)	0.031	0.26 (0.05, 0.47)	0.017
***Mood disorders***										
**Males**										
Mood disorders (*N =* 864)	3.64 ± 0.27	3.68 ± 0.27	3.69 ± 0.26	3.25 ± 0.26	−0.05 (−0.45, 0.36)	0.823	0.43 (0.02, 0.84)	0.038
No mood disorders (*N =* 5,031)	2.01 ± 0.23	1.71 ± 0.23	1.98 ± 0.23	1.60 ± 0.23	0.03 (−0.15, 0.21)	0.751	0.11 (−0.07, 0.29)	0.248
**Females**										
Mood disorders (*N =* 1,505)	5.06 ± 0.27	5.07 ± 0.26	4.59 ± 0.26	4.47 ± 0.25	0.47 (0.04, 0.49)	0.012	0.60 (0.24, 0.96)	0.001
No mood disorders (*N =* 4,654)	3.32 ± 0.25	3.06 ± 0.24	3.06 ± 0.25	2.78 ± 0.24	0.26 (0.10, 0.84)	0.020	0.27 (0.06, 0.49)	0.013

All models controlling for age, sex, BMI, income level, educational attainment, living status, smoking status, relationship status, and alcohol intake;

* Model also accounts for GAD-7 score at COVID-19 Survey entry.

α Significant (*p* < 0.05) difference between males and females at COVID-19 Survey entry.

β Significant (*p* < 0.05) difference between males and females at COVID-19 Survey exit.

## Discussion

Our study results suggest that pet ownership does not significantly promote mental well-being during the COVID-19 pandemic for people without mental health disorders; however, for people with mental health disorders, pet ownership may be associated with worse depressive and anxiety symptoms. Pet owners during the pandemic have experienced unique challenges, including: meeting the needs of their pets, securing supplies, accessing veterinary care, and dealing with new and emerging behavioral issues which may be related to changes in the daily routines (e.g., working from home) (23). Meeting these challenges does not appear to substantially impact the well-being of people without mental health disorders. However, for people with mental health disorders, the extra burden of care of an animal during the pandemic may contribute to poorer mental health outcomes.

Importantly, pet owners in our study were younger and more likely to have mental health disorders than non-owners. Mental health disorders are less prevalent in older adults than younger adults (24–26). Community-dwelling older adults also appear to be more resilient to the mental health effects of COVID-19 than younger adults (27). A history of mental health disorders is associated with a substantial increase in depressive and anxiety symptoms over and above pre-pandemic levels (6). Taken together, these attributes associated with pet ownership (i.e., younger age, pre-existing mental health disorders), as well as pet ownership itself, may create a perfect storm to negatively impact mental health during a pandemic.

Our sensitivity analyses suggest that there may be some sex differences in our results. Specifically, we determined that during the COVID-19 Survey that (1) females have greater anxiety and depressive symptoms than males, irrespective of mental health disorder status; and (2) pet ownership may have both positive and negative effects on mental health for females, but not for males. Females are twice as likely to be diagnosed with depression and most anxiety disorders, with the exception of bipolar disorder which has similar prevalence in males and females (28). While it is difficult to determine at this time why pet ownership appears to have had sex-specific effects on anxiety and depressive symptoms based on mental health disorder status, we think these sex differences should be explored further as the COVID-19 pandemic continues.

Our analysis used a large, longitudinal cohort of middle-aged and older Canadians who were queried about their mental health both before and during the COVID-19 pandemic. Nonetheless, there are several important limitations. We excluded participants with missing data. Our primary outcome currently does not have an established minimal clinically important difference (MCID) and thus it is difficult for us to determine what these differences in CESD-10 between pet owners and non-owners mean. Pet ownership status was based on a single question, and thus we cannot determine whether specific types of pets (e.g., dogs, cats, etc.) impact mental health differently. Ownership of pets was not queried during the COVID-19 Survey, and thus our estimates of pet ownership during the pandemic may be outdated and incorrect. It is also unclear whether different types of pets have differential effects on depressive/anxiety symptoms. Mental health status was determined by self-reported indication of a mental health disorder. Anxiety symptoms were not collected prior to the start of the COVID-19 pandemic. Finally, our sample was based on the CLSA cohort, who were aged 45 to 85 years at baseline. Thus, we did not include individuals who were younger than 45 or older than 85 years which may limit the generalizability of our results to younger or older populations.

In summary, our study suggests that during the COVID-19 pandemic pet ownership in people with mental health disorders is associated with poorer mental health outcomes compared with people with mental health disorders who do not own pets. By comparison, pet ownership is not associated with differences in anxiety or depressive symptoms in people without mental health disorders. Future work is still needed to understand the long-term effects of COVID-19 on mental health, and whether pets have benefits on mental health. More pandemics will inevitably occur in the future, and thus it is critical for research to continue on crisis-driven changes in human-animal relationships.

## CLSA team

Andrew Wister and Theodore Cosco (Simon Fraser University); Theone Paterson and Scott Hofer (University of Victoria); Verena Menec (University of Manitoba); Cynthia Balion and Laura Anderson (McMaster University); Mélanie Levasseur and Benoít Cossette (University of Sherbrooke); Christina Wolfson (McGill University); Phil St. John, Gerry Mugford, and Zhiwei Gao (Memorial University of Newfoundland); David Hogan (University of Calgary); Yukiko Asada (Dalhousie University).

## Data availability statement

The data analyzed in this study is subject to the following licenses/restrictions: Data are available from the Canadian Longitudinal Study on Aging (www.clsa-elcv.ca) for researchers who meet the criteria for access to de-identified CLSA data. Requests to access these datasets should be directed to www.clsa-elcv.ca.

## Ethics statement

The studies involving human participants were reviewed and approved by University of British Columbia's Clinical Research Ethics Board. The patients/participants provided their written informed consent to participate in this study.

## Author contributions

RF wrote the first draft of the manuscript and performed the statistical analysis. TL-A and MN wrote portions of the manuscript and provided key edits. TL-A, SK, LG, NB, JM, and PR designed the CLSA COVID-19 study and contributed to the revision of the manuscript. The members of the CLSA team have contributed to the collection of the data across Canada. All authors contributed to the article and approved the submitted version.

## Funding

Funding for the support of the CLSA COVID-19 Questionnaire based study is provided by Juravinski Research Institute, Faculty of Health Sciences, McMaster University, Provost Fund from McMaster University, McMaster Institute for Research on Aging, Public Health Agency of Canada and Government of Nova Scotia. Funding for the Canadian Longitudinal Study on Aging (CLSA) is provided by the Government of Canada through the Canadian Institutes of Health Research (CIHR) under grant reference: LSA 94473 and the Canada Foundation for Innovation, as well as the following provinces: Newfoundland, Nova Scotia, Quebec, Ontario, Manitoba, Alberta, and British Columbia.

## Conflict of interest

The authors declare that the research was conducted in the absence of any commercial or financial relationships that could be construed as a potential conflict of interest.

## Publisher's note

All claims expressed in this article are solely those of the authors and do not necessarily represent those of their affiliated organizations, or those of the publisher, the editors and the reviewers. Any product that may be evaluated in this article, or claim that may be made by its manufacturer, is not guaranteed or endorsed by the publisher.

## Author disclaimer

The opinions expressed in this manuscript are the author's own and do not reflect the views of the Canadian Longitudinal Study on Aging.
